# Draft Genome Sequences of *Bradyrhizobium elkanii* Strains BLY3-8 and BLY6-1, Which Are Incompatible with *Rj*_3_ Genotype Soybean Cultivars

**DOI:** 10.1128/genomeA.01169-16

**Published:** 2016-10-27

**Authors:** Aung Zaw Htwe, Yu Kanesaki, Hirofumi Yoshikawa, Hirohito Tsurumaru, Takeo Yamakawa

**Affiliations:** a Graduate School of Bioresource and Bioenvironmental Sciences, Faculty of Agriculture, Kyushu University, Hakozaki, Higashi-ku, Fukuoka, Japan; b NODAI Genome Research Center, Tokyo University of Agriculture, Setagaya, Tokyo, Japan; c Department of Bioscience, Tokyo University of Agriculture, Setagaya, Tokyo, Japan; d Faculty of Agriculture, Kagoshima University, Korimoto, Kagoshima, Japan; e Department of Biosciences & Biotechnology, Faculty of Agriculture, Kyushu University, Higashi-ku, Fukuoka, Japan

## Abstract

We report here the draft genome sequences of *Bradyrhizobium elkanii* strains BLY3-8 and BLY6-1, which are incompatible with *Rj*_3_ genotype soybean cultivars. The genome sequences of these strains will be useful to identify a causal gene for this incompatibility.

## GENOME ANNOUNCEMENT

*Bradyrhizobium elkanii* is known to have the ability to form nodules on soybean (*Glycine max*) root (1). However, nodulation of *B. elkanii* strains BLY3-8 and BLY6-1 is restricted by soybean cultivars CNS (2) and D51 (our unpublished data). This restriction is due to the *Rj*_3_ gene in soybeans (3, 4). In order to aid in identification of the gene causing this incompatibility in strains BLY3-8 and BLY6-1, we conducted genome sequencing of these strains.

Genomic DNA of *B. elkanii* strains BLY3-8 and BLY6-1 was extracted using the ISOPLANT kit (Nippon Gene, Tokyo, Japan) and fragmented to 400 bp using a Covaris S2-A (Covaris, Woburn, MA). The libraries for sequencing were prepared by a NEBNext DNA library prep master mix set for Illumina (New England BioLabs, Ipswich, MA) and paired-end sequenced (2 × 300 bp) on a MiSeq sequencer using a MiSeq reagent kit version 3 (Illumina KK, Tokyo, Japan). Raw reads were trimmed and *de novo* assembled using CLC Genomics Workbench 7.5 (Qiagen, Valencia, CA). Parameters for the trimming were as follows: ambiguous limit, 0; quality limit, 0.01; number of 5′ terminal nucleotides, 10; number of 3′ terminal nucleotides, 14; minimum length, 150 bp. The default settings were used for the *de novo* assembly parameters (i.e., mapping mode, map reads back to contigs [slow]; mismatch cost, 2; insertion cost, 3; deletion cost, 3; length fraction, 0.8; similarity fraction, 0.9).

The draft genomes of *B. elkanii* strains BLY3-8 and BLY6-1 were assembled into 90 and 79 contigs, respectively. The accumulated lengths of these genomes were 9,198,916 bp (*N*_50_, 302,740 bp) and 9,202,892 bp (*N*_50_, 363,283 bp), respectively. The G+C content of both genomes was 63.8%. The total numbers of coding sequences (CDSs) predicted by the Prokaryotic Genome Annotation Pipeline (PGAP 3.1) were 8,036 and 8,049, respectively.

### Accession number(s).

The whole-genome shotgun projects for *B. elkanii* strains BLY3-8 and BLY6-1 have been deposited at DDBJ/EMBL/GenBank under the accession numbers LWUI00000000 and LXEM00000000, respectively. The versions described in this paper are versions LWUI01000000 and LXEM01000000, respectively.
